# X-linked Inhibitor of Apoptosis (XIAP) Deficiency Complicated by Hemophagocytic Lymphohistiocytosis on Immunotherapy Leading to Acute Respiratory Distress Syndrome and Multiorgan Failure Secondary to Opportunistic Infections

**DOI:** 10.7759/cureus.62703

**Published:** 2024-06-19

**Authors:** David Kim, Stephanie Liu, Eli Zolotov, Roma Padalkar

**Affiliations:** 1 Internal Medicine, Hackensack University Medical Center, Hackensack, USA; 2 Family Medicine, JFK University Medical Center, Edison, USA

**Keywords:** pjp, mai, ards, hemophagocytic lymphohistiocytosis, xiap deficiency

## Abstract

X-linked inhibitor of apoptosis (XIAP) deficiency is a rare primary immunodeficiency with a broad spectrum of clinical manifestations, including susceptibility to hemophagocytic lymphohistiocytosis (HLH), inflammatory bowel disease (IBD), hypogammaglobulinemia, and severe infections. We present a case of a 39-year-old male with a past medical history of XIAP deficiency complicated by HLH, Crohn’s disease, and hypogammaglobulinemia, who developed acute respiratory distress syndrome (ARDS) due to Pneumocystis jiroveci pneumonia (PJP) and concurrent multiorgan failure due to disseminated Mycobacterium avium intracellulare (MAI) infection. This case highlights the challenges in managing XIAP deficiency, emphasizing the importance of early recognition, and the need for further research to improve outcomes in this population.

## Introduction

X-linked inhibitor of apoptosis (XIAP) deficiency is a rare primary immunodeficiency caused by mutations in the XIAP/BIRC4 gene that is estimated to occur in 1-2 per million live male births [1-3]. Given the novelty of its recognition, the pathophysiology of XIAP deficiency is not yet fully understood. However, the XIAP protein is believed to be involved in immune responses and regulation through tumor necrosis factor receptor signaling [1]. It is characterized by a broad spectrum of clinical manifestations, most notable for its high susceptibility to recurrent hemophagocytic lymphohistiocytosis (HLH), inflammatory bowel disease (IBD), hypogammaglobulinemia, and severe recurrent infections [3,4]. The gold standard for diagnosing XIAP deficiency is genetic sequencing to identify the disease-causing mutations in the XIAP/BIRC4 gene [1]. Currently, there are no established guidelines for treating XIAP deficiency and the only known curative therapy is allogeneic hematopoietic stem cell transplantation (HSCT), albeit with lower survival probabilities compared to the population eligible to be treated conservatively [5].

This case represents the first reported case of XIAP deficiency leading to a cascade of clinical manifestations, which ultimately results in mortality secondary to acute respiratory distress syndrome from Pneumocystis jiroveci pneumonia and multiorgan failure in the setting of fulminant Mycobacterium avium intracellulare (MAI) infection.

## Case presentation

The patient was a 39-year-old male with a complex medical history, including XIAP deficiency complicated by HLH for which he was receiving immunotherapy. He also had hypogammaglobulinemia treated with intravenous immune globulin (IVIG) and a history of Crohn's disease since childhood, for which he underwent ileostomy and total colectomy.

The patient was admitted five days before this admission due to a three-day history of worsening shortness of breath. Diagnostic tests revealed a sputum culture positive for Pseudomonas aeruginosa (treated with a five-day course of piperacillin/tazobactam), a polymerase chain reaction (PCR) test positive for PJP, and a bronchial alveolar lavage (BAL) culture positive for Candida glabrata. Additionally, the patient developed cutaneous nodules on the upper and lower extremities, raising suspicion of other opportunistic infections. The biopsy was later positive for acid-fast bacilli, indicating MAI infection.

Treatment for MAI was initiated with azithromycin, rifampin, and ethambutol. His immunosuppressive therapy was adjusted, reducing anakinra from 200 mg twice daily to 100 mg twice daily, and dexamethasone from 10 to 6 mg daily, following the immunology team’s recommendations.

**Figure 1:**
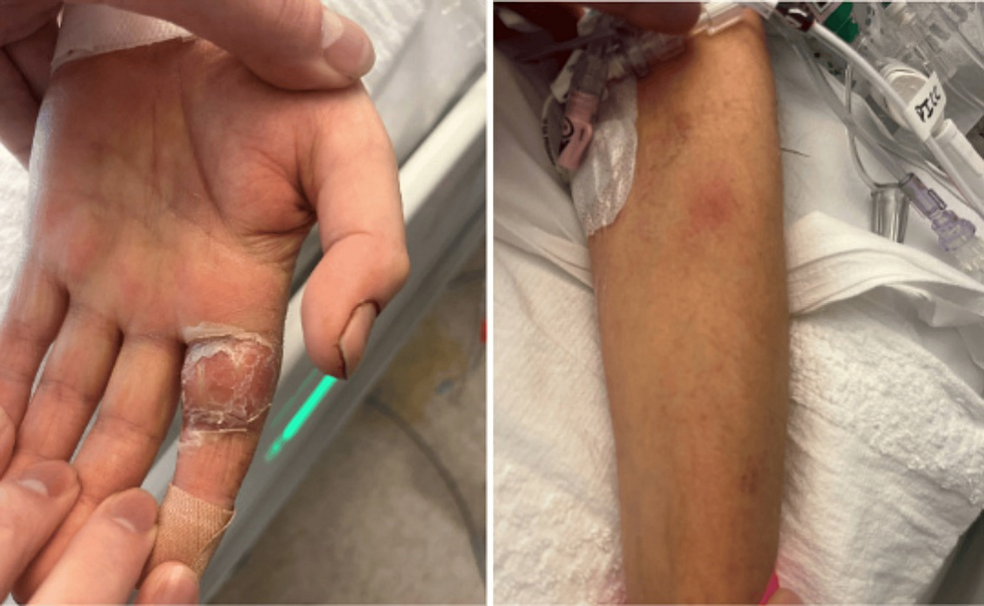
Multiple skin nodules on both upper and lower extremities, with the most prominent swelling and erythema observed on the proximal phalanx of the left index finger.

**Figure 2:**
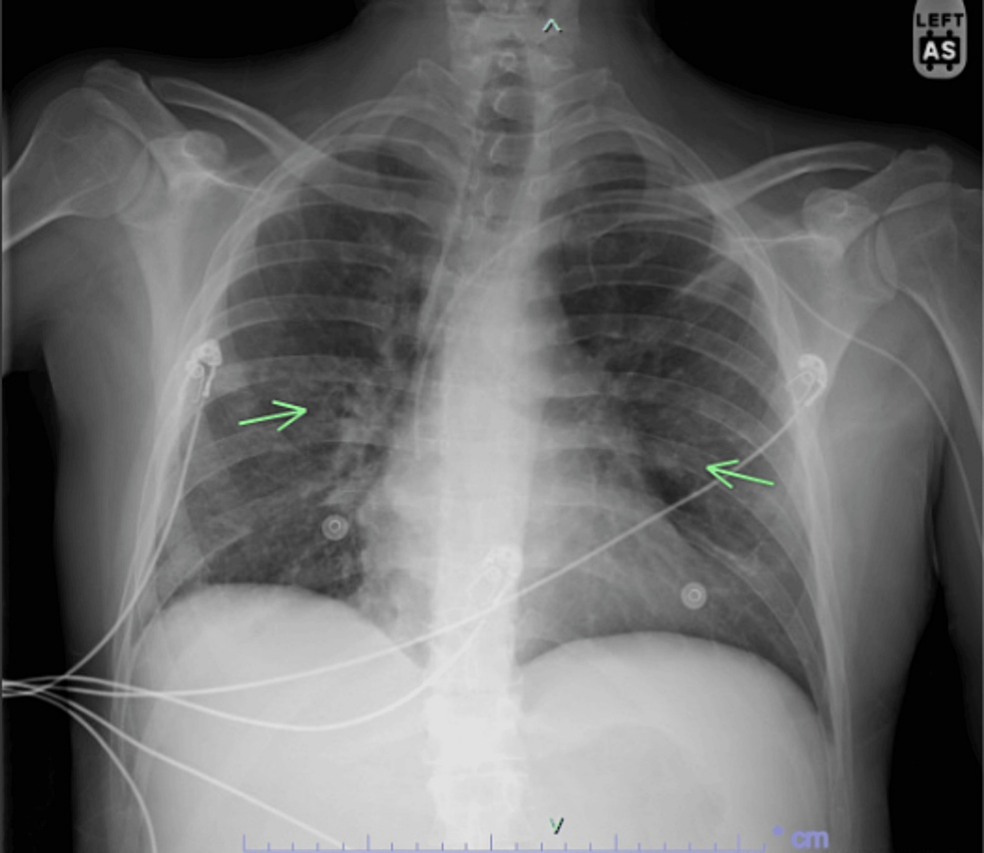
Initial chest X-ray showing residual mild interstitial prominence bilaterally, as indicated by the arrows.

The patient was readmitted five days later with worsening shortness of breath. On admission, the patient was hypotensive with a blood pressure of 96/60 mmHg, tachycardic with a heart rate of 111 beats/minute, tachypneic with a respiratory rate of 23 breaths/minute, and saturating at 95% on 3 L of oxygen via nasal cannula. Physical examination noted multiple skin nodules with prominent swelling and erythema of the proximal phalanx of the left index finger, which were biopsy-positive for acid-fast bacteria (Figure 1). Initial laboratory results showed worsening pancytopenia, lactic acidosis, mild electrolyte derangements with acute kidney injury, and transaminitis, as summarized in Table 1. On initial imaging, chest X-ray was significant for mild residual interstitial prominence bilaterally (Figure 2). The patient was started on a sepsis protocol with intravenous fluids and an extensive antibiotic regimen, including azithromycin, levofloxacin, and amikacin, for MAI infection and bactrim for PJP. Throughout the hospital course, extensive multidisciplinary discussions were conducted to optimize the medical therapy regimen with underlying XIAP deficiency and its complications in the setting of sepsis.

**Table 1:** Laboratory data on recent prior admission and current admission.

Variable	Prior admission	Admission	Reference range
Hemoglobin (g/dL)	8.9	7.1	13.0-17.0
Hematocrit (%)	26	19.5	36-46
White-cell count (per μL)	3,400	500	4,000-11,000
Neutrophils (%)	72	75	40-75
Lymphocytes (%)	6	11	13-43
Monocytes (%)	11	4	0-13
Eosinophils (%)	1	0	0-5
Platelet count (per μL)	71	52	135,000-430,000
Sodium (mmol/L)	132	130	136-145
Potassium (mmol/L)	4.4	3	3.5-5.1
Carbon dioxide (mmol/L)	23	20	22-29
Creatinine (mg/dL)	1.09	1.26	0.3-1.5
Aspartate aminotransferase (U/L)	42	58	5-34
Alanine aminotransferase (U/L)	34	34	0-55
Total bilirubin (mg/dL)	1.1	7.2	0.2-1.2
Alkaline phosphatase (IU/L)	173	327	43-122
Glucose (mg/dL)	133	67	82-115
Lactate (mmol/L)	0.8	3	0.5-2.0

After consulting the immunology team, his immunotherapy with anakinra was increased from 100 mg twice daily to 200 mg three times daily, dexamethasone was maintained at 10 mg twice daily, and ruxolitinib (15 mg twice daily) was added due to worsening pancytopenia, which was presumed to be secondary to HLH. 

**Figure 3:**
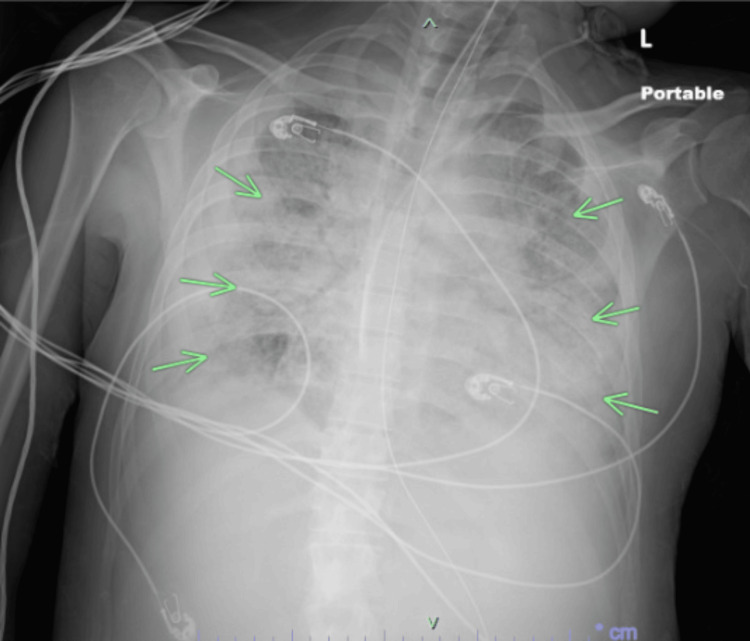
Chest X-ray post-intubation that showed significant worsening of bilateral infiltrative disease (as emphasized by the arrows), indicative of acute hypoxic respiratory distress.

The hospital course was complicated by altered mental status and hypoxia, necessitating emergent intubation. Repeat chest X-rays indicated progressively worsening infiltrates, consistent with ARDS (Figure 3). Subsequent bronchial alveolar lavage revealed mold growth. The patient’s renal function continued to decline, necessitating continuous renal replacement therapy in the intensive care unit. Additionally, he developed a small bowel obstruction, which required nasogastric tube placement. Despite consultation with the surgery team, surgical intervention was deemed inappropriate due to the patient's overall deteriorating clinical condition.

Ultimately, the patient continued to deteriorate with septic shock, presumed to be from multiple opportunistic infections. He required vasopressors and maximum ventilator support for ARDS, but his condition did not improve. After extensive discussion, the family elected to transition the patient to comfort care. The patient was terminally extubated and passed away.

## Discussion

Currently, there are no established guidelines for diagnosing and treating XIAP deficiency. The gold standard for diagnosing XIAP deficiency is genetic sequencing [1]; however, the challenge remains in early recognition of the disease and subsequent testing for diagnosis as it is a novel and rare disease. Apart from HSCT, the current therapeutic approach includes a multimodal treatment plan to individually address the associated disease manifestations, such as chemotherapy and/or corticosteroids for HLH, immunoglobulin replacement therapy for hypogammaglobulinemia, immunosuppressive therapy, and/or surgical approach for IBD [1]. There have been very few studies conducted in the past decade on the patient population with the disease who underwent HSCT. Early studies have indicated poor outcomes, with complications arising from transplant-related toxicities such as vaso-occlusive diseases and pulmonary hemorrhage [6-7]. Given the novelty of the disease, our options for treatment of the patients with XIAP deficiencies are greatly limited when they present with acute manifestations of the disease.

Managing patients with XIAP deficiency is particularly challenging when they present with ambiguous signs, such as worsening pancytopenia, which may be due to HLH secondary to XIAP deficiency or a phenotype secondary to sepsis [8]. Identifying the primary cause is crucial for optimizing medical therapy, as one path requires increasing immunotherapy, while the other benefits from reducing it. In our case, reducing immunosuppressive therapy due to opportunistic infections may have worsened HLH, leading to neutropenia and further infections. However, without initial intervention, the patient would have been further immunocompromised, increasing the risk of other opportunistic infections. Moreover, HLH itself also is a complicated manifestation of XIAP deficiency without in-depth studies in the adult population. Current guidelines to diagnose and treat HLH are heavily reliant on studies based on the pediatric population, which may result in overtreatment and toxicity in adults [9-10].

This is the first reported case depicting the acute complications of XIAP deficiency leading to mortality. In our case, the patient expressed a wide spectrum of clinical manifestations of XIAP deficiency, including HLH, severe infections, and small bowel obstruction (SBO) secondary to MAI infection in the setting of prior IBD, which made the hospital course extremely challenging. Given the limitations of treating its acute clinical manifestations, further studies are warranted to understand and discover new approaches for earlier diagnosis and treatment of XIAP deficiency to prevent the cascade of clinical manifestations that lead to mortality in this population.

## Conclusions

In conclusion, this case highlights the complex and potentially life-threatening nature of XIAP deficiency, particularly when complicated by an immunosuppressed state, leading to opportunistic infections. The lack of established guidelines for the management of XIAP deficiency underscores the importance of further research to elucidate its pathophysiology and identify effective treatment strategies. This case also emphasizes the need for heightened clinical suspicion of XIAP deficiency in patients presenting with recurrent infections and inflammatory manifestations. Early recognition and multidisciplinary management are crucial in optimizing outcomes for patients with XIAP deficiency and preventing fatal complications such as acute respiratory distress syndrome and multiorgan failure.
